# Molecular ecology and selection in the drought-related *Asr* gene polymorphisms in wild and cultivated common bean (*Phaseolus vulgaris* L.)

**DOI:** 10.1186/1471-2156-13-58

**Published:** 2012-07-16

**Authors:** Andrés J Cortés, M Carolina Chavarro, Santiago Madriñán, Dominique This, Matthew W Blair

**Affiliations:** 1 Departamento de Biologia, Universidad de los Andes, Carrera 1 N° 18A – 12, J302, Bogotá, Colombia; 2 Department of Agronomy, University of Georgia, 111 Riverbend Road, Athens, GA, USA; 3 Montpellier SupAgro, UMR AGAP, TA-A 108/03, Ave Agropolis, 34398, Montpellier, cedex 5 - France; 4 Department of Plant Breeding and Genetics, Cornell University, 242 Emerson Hall, Ithaca, NY, United Sates; 5 Department of Evolutionary Biology, Uppsala University, Uppsala, Sweden

## Abstract

**Background:**

The abscisic acid (ABA) pathway plays an important role in the plants’ reaction to drought stress and ABA-stress response (*Asr*) genes are important in controlling this process. In this sense, we accessed nucleotide diversity at two candidate genes for drought tolerance (*Asr1* and *Asr2*), involved in an ABA signaling pathway, in the reference collection of cultivated common bean (*Phaseolus vulgaris* L.) and a core collection of wild common bean accessions.

**Results:**

Our wild population samples covered a range of mesic (semi-arid) to very dry (desert) habitats, while our cultivated samples presented a wide spectrum of drought tolerance. Both genes showed very different patterns of nucleotide variation. *Asr1* exhibited very low nucleotide diversity relative to the neutral reference loci that were previously surveyed in these populations. This suggests that strong purifying selection has been acting on this gene. In contrast, *Asr2* exhibited higher levels of nucleotide diversity, which is indicative of adaptive selection. These patterns were more notable in wild beans than in cultivated common beans indicting that natural selection has played a role over long time periods compared to farmer selection since domestication.

**Conclusions:**

Together these results suggested the importance of *Asr1* in the context of drought tolerance, and constitute the first steps towards an association study between genetic polymorphism of this gene family and variation in drought tolerance traits. Furthermore, one of our major successes was to find that wild common bean is a reservoir of genetic variation and selection signatures at *Asr* genes, which may be useful for breeding drought tolerance in cultivated common bean.

## Background

Common bean (*Phaseolus vulgaris* L.) is the most important food legume in terms of providing directly consumed nutrients and dietary protein in developing countries of Latin America, Africa as well as in traditional diets of the Middle East and the Mediterranean with over 23 M tons grown around the tropics, sub-tropics and temperate zones for anywhere from on-farm to local market, and within-country consumption or exports [1]. Common bean is usually grown in areas with sufficient rainfall but has also extended to regions where drought is endemic and supplemental irrigation is scarce such as in northeastern Brazil, coastal Peru, the central and northern highlands of Mexico, and in lower elevations of East Africa [2] as well as the western plains of the United States and Canada [3]. Therefore, increasing drought tolerance through common bean breeding has become a common goal of national and international breeding programs.

Relatively few sources of drought tolerance have been identified in common bean compared to other legumes. Most studies have concentrated on advanced lines from a few commercial classes [4] and not from the wild and landrace collections which are considerable reservoirs of naturally-adapted genotypes for drought-stress environments. Therefore, searching wild and cultivated collections of common bean is another goal of plant breeding programs and has recently been assigned a high level of funding within the context of the food security programs of the International Agricultural Research Centers. Initial testing has shown high variability for drought tolerance traits and certainly common bean contains a lot of allele richness [5,6].

Drought tolerance is a genetically, physiological and mechanistic complex trait. In terms of genetics, the multiple individual traits that make up drought tolerance are usually inherited quantitatively with very few major genes for drought tolerance mechanisms known, although Blair *et al.*[7] did find some quantitative trait loci for drought tolerance. Epigenetic and environmental components of drought stress exist, as well. One transcription factor that is often involved in signaling of drought stress is abscisic acid whose levels are often correlated with plant parts and whole plants that are suffering from drought stress [8]. Some of the mechanisms of drought tolerance are controlled through an ABA responsive pathway [9], while others are independent of ABA [8,10]. In particular, the transcription factors of the *Asr* (abscisic acid, stress, ripening induced) family of genes are plant-specific and stress-regulated components of the ABA- dependent pathway, with further proof of their role found in their interaction with ABRE elements [11,12]. Sucrose synthase genes are thought to be downstream *Asr* genes [12]. The number of *Asr* genes found in plant genome databases varies from one in *Vitis vitifera*, four in *Brachypodium distachyon*, six in *Oryza sativa*, and up to seven in *Sorghum bicolor*[13]. Expression analysis have demonstrated their explicit role in conferring increased drought and salt tolerance in tomato, rice and lilies [14-16] but to date no analysis of their role in the legumes has been put forward.

In this regards, diversity analysis of the ASR family has been illustrative of adaptive selection of crop plants to help deal with environmental conditions. For example, studies of the extent of nucleotide diversity in *Asr* genes in Solanaceae species and in wild and cultivated rice provided some evidence of non-neutral evolution, adaptive, or demographic events in dry areas [14,17,18]. Moreover, genetic mapping of *Asr1* co-localized this gene with QTLs for xylem sap ABA content, for anthesis–silking interval responsive to mild water deprivation and for leaf senescence in maize [19,20]. Finally, Maskin *et al.*[21] showed a DNA-binding activity, Konrad & Bar-Zvi [22] revealed that the unstructured form of tomato ASR1 proteins presented a chaperone-like activity that stabilized other proteins against denaturation caused by heat and freeze–thaw cycles, and Cakir *et al.*[23] described an association with a grape hexose transporter promoter.

Common bean is a good model to study drought related candidate genes and especially the less complex *Asr* family because of its rich evolutionary history in the wild across two continents (South and Central America) and multiple domestication process (in the Andean and Mesoamerican regions of the Americas). Wild *Phaseolus vulgaris* beans are diverse in the western hemisphere ranging from temperate Argentina to dry land parts of Mexico. However, wild beans are thought to have evolved from an original gene pool in Ecuador and northern Peru, after which radiation to various regions north and south of there, gave rise to an Andean, a Colombian, and a Mesoamerican gene pool [24]. The Andean and Mesoamerican wild beans were then subjected to domestication in each region giving rise to cultivars of both gene pools [25-27].

Mesoamerican beans were domesticated in the region of Jalisco [28], although this does not preclude more than one domestication event in another part of Mesoamerica giving rise to a diversity of chloroplast haplotypes [29]. For the Andean gene pool, southern Bolivia may have been the center of domestication [30] with introgression from the wild occurring in the extension of cultivated types northwards towards the equator. Both domestications occurred 5,000–8,000 years ago [31]. Therefore in common bean, populations structure is divided into gene pools (Andean and Mesoamerican for cultivated beans and four or more groups in wild beans); while additional structure within each of these gene pools is then found. Within the cultivated Andean gene pool the races Nueva Granada, Peru and Chile are identifiable [5,32-35]. Within the cultivated Mesoamerican gene pool the race Mesoamerica, the complex Durango-Jalisco and the race Guatemala are observable [5,36].

As mentioned above, the cultivated gene pool structure contrasts with the structure obtained for wild common bean in which four main clusters are seen: the Colombo-Mesoamerican, the Mexican, the Andean and the Peruvian-Ecuadorian [24]. This simplistic model has been further challenged by results from Kwak *et al.*[33] where wild common beans from Central America were divisible into different groups including those from Guatemala and Costa Rica.

Introgression between gene pools, between the races and between cultivated and wild genotypes has been a historical, long-term and re-iterative process [34,37,38]. However, the structure of the wild populations is maintained by geographic barriers along the length of the Andean to Mesoamerica arc of mountains and varying terrain. In terms of habitat ecology, race Durango-Jalisco is the only one of the groups of races that has significant drought tolerance, with part of this group distributed in semi-arid areas of Mexico [39]; race Chile has adaptation to relative drier areas as well, but is only found in the southern Andes [35,40]. Races Mesoamerica and Guatemala, or Nueva Granada and Peru occupy low to mid altitude or highland regions of Latin America, respectively [5,41,42]. Some highland and mid-elevation sites are also drought susceptible especially in the equatorial regions where bimodal rainfall events are often short. Although cultivars from the Durango-Jalisco complex have the highest level of drought tolerance, one may expect to find still higher levels in certain wild germplasm [2].

Nucleotide diversity surveys are powerful tools for the study of reference collections of cultivated and wild genotypes that allow population genetic tests to be made for departure from the neutral equilibrium models and to identify the diverse selective modes that shaped the evolution of specific genes [43]. For instance, sequence information for specific genes can show their genealogy and suggest how different groups of accessions evolved from the wild and how this influenced the genotypes involved in domestication events. Therefore, the particular role of duplication, lineage sorting, sub-functionalization, sub-speciation and ecological constrains on gene evolution can be inferred. Furthermore, population structure, SNP diversity and phenotype association are other activities which can we undertaken [44].

The specific goals of this research were to evaluate the allele diversity of the *Asr1* and *Asr2* genes in wild and cultivated common bean and to determine (1) the extent of haplotype diversity, (2) the allele distribution in relation with the gene pool origins and probable drought tolerance based on geographic origin, (3) the differences at these candidate genes between wild and cultivated common beans, and (4) the patterns of nucleotide variation as related to local adaptation to ecological environments.

## Results

### Structure of the ASR family

**Figure 1:**
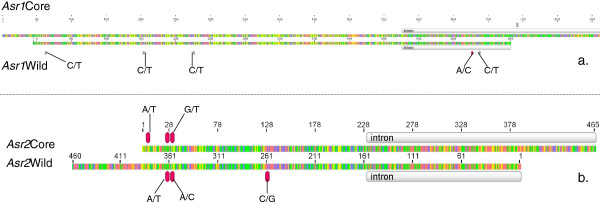
**Genetic regions considered for the diversity analysis of *Asr1 *and *Asr2.*** Genetic regions considered for the diversity analysis of **a**. *Asr1* and **b**. *Asr2* in the wild and cultivated collections (Core). Silver boxes are introns. Gray markers are transitions and pink markers are transversions.

There are two members of the ASR gene (PF02496) in common bean that could be distinguished by the primers we used here. Both of them have two exons and one intron. The *Asr2* gene is smaller than the *Asr1* gene. *Asr2* includes 100 bp upstream of the ABA domain whilst *Asr1* includes 550 bp. In addition, the intron in *Asr2* (179 bp) is smaller than the intron in *Asr1* (565 bp) (Figure 1). This structure was confirmed with the alignment between common bean ESTs (TC2798 and CA910244) and our sequenced region.

### Polymorphism and deviation from neutrality at the *Asr* genes

Molecular variation in the wild bean collection was significantly structured and all analyzed regions of both genes were related to the sub-population structure except for *Asr2*. The *G*_*st*_, *F*_*st*_ and Snn values were 0.344, 0.495 and 0.256 for *Asr1*, and 0.167, 0.426 and 0.350 for *Asr2*, showing the higher differentiation in the first gene compared to the second gene. Recombination parameters per gene [45] and minimum number of recombination events using the four-gamete test were higher for the intron region of each gene than for the exon regions of the genes (0.8 and 0.001 respectively). In general, *Asr* genes presented low polymorphism as reflected by PIC (polymorphic information content) values for SNPs, although these were higher on average for *Asr2* than for *Asr1*, and much higher in the wild collection than in the cultivated collection as would be expected by a domestication bottleneck. Polymorphic sites were generally more common in introns and non-conserved domains than in exons or conserved regions (Table 1).

**Table 1:** Genetic diversity values for SNPs across the 401 accessions of the common bean wild (104 accessions) and cultivated (297 accessions) collections

**Gene**	**Set of sequences**	**SNP**	**Position consensus**	**SNP type**	**Majority allele**	**Minority allele**	**Majority allele frequency**	**Minority allele frequency**	PIC*
*Asr1*	Cultivated	1	621	[T/C]	T	C	0.56	0.44	**0.49**
	Wild intron	1	57	[A/C]	A	C	0.91	0.09	*0.17*
		2	68	[T/C]	T	C	0.88	0.12	**0.21**
	Wild exon	1	20	[T/C]	C	T	0.97	0.03	0.05
		2	161	[T/C]	T	C	0.81	0.19	**0.31**
		3	233	[T/C]	C	T	0.84	0.16	**0.27**
*Asr2*	Total**	1	24	[A/T]	T	A	0.86	0.14	**0.25**
		2	29	[T/G]	G	T	0.86	0.14	**0.25**
		3	127	[C/G]	C	G	0.98	0.02	0.03
	Cultivated	1	6	[A/T]	A	T	0.93	0.07	*0.12*
		2	26	[A/T]	T	A	0.93	0.07	*0.12*
		3	31	[T/G]	G	T	0.93	0.07	*0.12*
	Wild	1	247	[C/G]	G	C	0.93	0.07	*0.13*
		2	345	[A/C]	C	A	0.62	0.38	**0.47**
		3	350	[A/T]	A	T	0.62	0.38	**0.47**

* PIC values higher than 0.2 indicated in bold text, PIC values between 0.1 and 0.19 indicated in italics.**Polymorphic sites only observed in heterozygotes are not depicted.

In total there were similar numbers of SNPs in each gene, however in *Asr1* more were in the wild accessions compared to *Asr2* where the SNPs were equally divided between cultivated and wild. Interestingly, all mutations in coding regions of *Asr1* and *Asr2* genes were synonymous mutations. Additionally, *Asr2* polymorphic sites distinguished DOR364 from the other three cultivars used as controls, while *Asr1* did not distinguish between Andean or Mesoamerican controls, although Tajima’s *D* varied between the gene pools (Table 2).

**Table 2:** **Diversity analysis for *Asr1 *and *Asr2 *genes in the wild and cultivated collections evaluated in this study **

**Gene**	**Region**	**Nb SNPs**	**Nb alleles**	**Watterson’s theta**	**Pi**	***D*Tajima**	**p-value*D***_**T**_	***D*Tajima (Andean)**	**p-value D**_**A**_**(Andean)**	***D*Tajima (Mesoamerican)**	**p-value D**_**M**_**(Mesoamerican)**	**Nb haplotypes**	**Heterozygosity**
*Asr1*	Cultivated	1	234	0.0002	0.0005	1.9518	NS	1.2539	NS	1.8516	NS	2	0.492
	Wild exon	3	74	0.0021	0.0022	0.0751	NS	−0.22	NS	0.3894	NS	3	0.316
	Wild intron	2	66	0.0037	0.0034	−0.1437	NS	−0.16	NS	0.1730	NS	3	0.356
*Asr2*	Total	3	576	0.0016	0.0021	0.5497	NS	0.1918	NS	1.0392	NS	4	0.257
	Cultivated	3	576	0.0016	0.0021	0.5497	NS	−0.4313	NS	−0.7107	NS	4	0.125
	Wild	3	146	0.0018	0.0040	2.1836	**<0.001**	0.7739	NS	2.3545	NS	4	0.533

Nb: Number of polymorphic sites, alleles or haplotypes.

NS: Non significant.

Deviations from Wright-Fisher neutrality were significant in the wild collection for *Asr2* when the analysis was carried out on a global basis, without considering population structure, and for *Asr1* when the analysis was carried out taking into account Mesoamerica and Guatemala populations (Table 3). Tajima’s *D* was positive in the first case and negative in the second one. The number of polymorphic sites found in Mesoamerican and Guatemala populations was higher than that found in Ecuador-Northern Peru and Andean populations. Moreover, no significant deviations were observed in other populations or gene pools. Ramos-Onsins & Rozas‘R2 values, which test for population growth, were not significant except for the Mesoamerican wild population.

**Table 3:** Details of nucleotide diversity and neutrality tests for candidate genes in the wild collection

**Gene**	**Set**	**Population**	**P(C)**	**h**	**S**	**Ts**	**Tv**	**Ka**	**Ks**	**ω**	**π (E**^**-3**^**)**	**S.D. π (E**^**-3**^**)**	**θ**_**w**_**(E**^**-3**^**)**	**S.D. θ**_**W**_**(E**^**-3**^**)**	**TD**	**DT 95% C.I.**		**p-value D**_**T**_	**R**^**2**^	**p-value R**^**2**^	**Max. K**
*Asr 1*	Exon	**Global**	**0.95**	**3**	**3**	**3**			**3**	**0.0**	**2.2**	**0.6**	**2.4**	**1.5**	**−0.22**	**−1.72**	**2.07**	**0.47**	**1.1**	**0.46**	**2**
		M	0.85	3	3	3			3	0.0	2.2	1.1	3.4	2.2	−1.18	−1.45	1.72	0.08	1.3	**<0,001**	2
		G	0.75	2	2	2			2	0.0	1.9	1.3	2.8	2.1	−1.24	−1.24	1.65	**<0,001**	3.5	0.87	2
		E	0.67	2	2	2			2	0.0	2.7	1.6	3.3	2.6	−0.97	−0.97	1.46	0.46	4.0	0.85	2
	Intron	**Global**	**0.95**	**3**	**2**	**1**	**1**				**3.9**	**1.0**	**4.2**	**3.1**	**−0.16**	**−1.49**	**2.09**	**0.48**	**1.1**	**0.36**	**2**
		M	0.86	2	1		1				4.1	1.0	2.9	2.9	0.95	−1.46	1.48	0.68	2.3	0.33	1
		G	0.75	2	1		1				2.5	1.7	3.6	3.6	−1.01	−1.01	1.34	0.52	3.5	0.50	1
*Asr 2*	Global	**Global**	**0.97**	**4**	**3**		**3**		**1**	0.0	**4.1**	**0.3**	**2.6**	**1.2**	**2.18**	**0.97**	**−1.62**	**<0,001**	**2.0**	**0.97**	**4**
		M	0.94	3	3		3		1	0.0	4.0	0.4	2.5	1.4	1.43	0.94	−1.74	1.92	1.9	0.92	4
		G	0.78	2	3		3				4.1	1.0	3.0	2.0	1.60	0.91	−1.45	1.73	2.7	0.85	3
		C	0.78	3	3		3				4.3	0.7	3.0	2.0	1.85	0.99	−1.45	1.73	2.8	0.93	3
		A	0.91	3	3		3				2.5	0.9	2.8	1.4	0.48	0.72	−1.73	2.02	1.6	0.69	3
	Exon	**Global**	**0.97**	**2**	**1**		**1**		**1**	0.0	**0.7**	**0.3**	**1.1**	**1.1**	**−0.46**	**0.49**	**−1.06**	**1.78**	**0.6**	**0.28**	**1**
		M	0.94	2	1		1		1	0.0	0.3	0.3	1.4	1.4	−1.14	0.28	−1.14	1.64	1.7	0.69	1
	UTR	**Global**	**0.97**	**3**	**2**		**2**				**23.1**	**1.3**	**9.8**	**6.0**	**1.61**	**0.99**	**−1.57**	**1.96**	**2.4**	**0.99**	**3**
		M	0.94	2	2		2				23.7	1.9	11.8	7.4	2.32	0.99	−1.73	2.14	2.5	0.99	3
		G	0.78	2	2		2				25.5	5.9	18.4	12.4	1.60	0.92	−1.45	1.86	2.7	0.88	3
		C	0.78	2	2		2				26.6	4.2	18.4	12.4	1.85	0.98	−1.45	1.73	2.8	0.93	3
		A	0.91	2	2		2				15.7	5.3	17.7	8.4	0.48	0.73	−1.73	1.87	0.16	0.65	0.03

h: number of haplotypes, S: number of polymorphic sites.

Populations M: Mesoamerican, G: Guatemala, C: Colombia, E: Peru and Ecuador, A: Andean.

P(C): Probability of having captured the deepest coalescent event.

π: nucleotide diversity Ts: Transitions, Tv: Transversions, ω: Non synonymous, synonymous substitution ratio (Ka/Ks) (Only for protein coding regions).

TD: Tajima’s *D*, θ_w_: Theta of Watterson (per site, from S), S.D.: Standard deviation.

p-Value: probability estimator ≠ 0 as determined by coalescent simulation.

R^2^: Ramos-Onsins & Rozas’ R^2^ (tests population growth), Max. K: Maximum number of nucleotide differences between any two sequences.

Empty cells: No applicable data (when h = 0).

**Figure 2:**
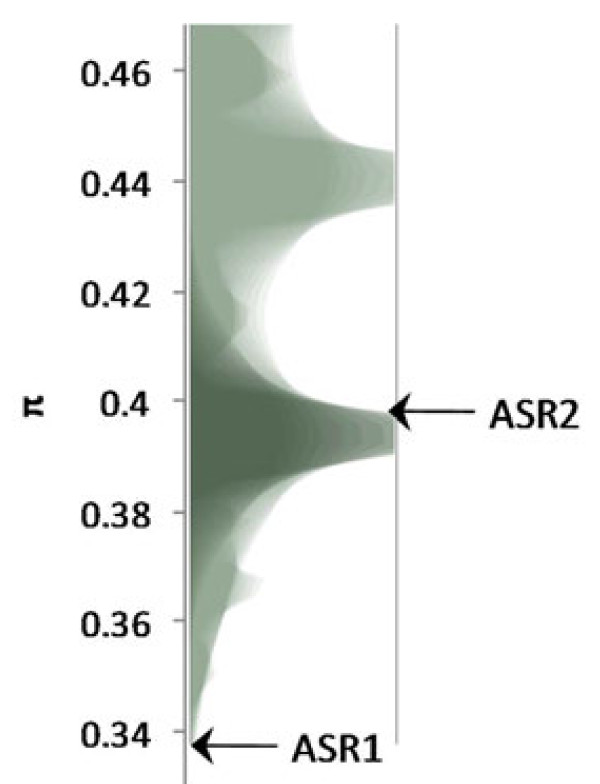
**Comparison of *Asr *genes to *Phaseolus vulgaris *general nucleotide diversity.** π (E-^2^)statistic from the wild collection was computed. The background distribution was estimated by Cortés *et al.*[45]. *Asr1* and *Asr*2 are indicated by arrows in the corresponding classes. Population structure and adaptive selection are associated with a high π values, but bottlenecks and directional selection is associated with low π value.

π values for *Asr1* and *Asr2* were compared against the background distribution from Cortés *et al.*[45] for non-drought related genes. Despite this selection of random genes, the background distribution was not neutral and tended to inflate π values, as would be expected where some of the random genes were important for sub-population selection. *Asr2* fell within the range of random genes. However, *Asr1* presented extreme lower values in relation with the evolutionary background, accounting for a p-value less than 0.001 (Figure 2). This shows that like the background distribution of SNPs, the present SNPs accounted for the gene pool structure.

### Haplotype analysis

**Figure 3:**
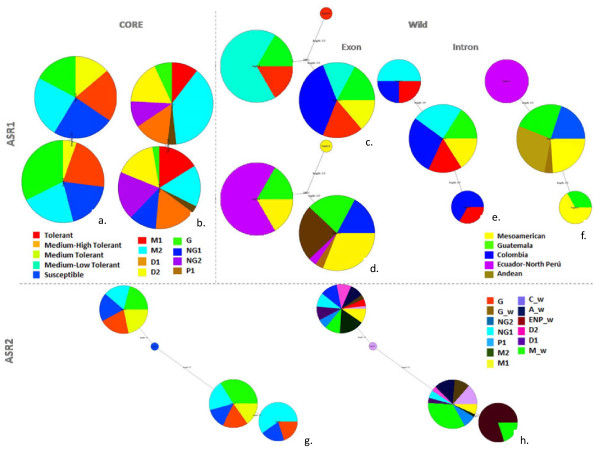
**Haplotype networks for*Asr1 *and *Asr2.*** Haplotype networks for *Asr1* and *Asr2* (subfigures a-f and g-h, respectively). Each node represents a haplotype, its size being proportional to its frequency. A segment corresponds to a subset of substitutions. Hollow nodes are hypothetical" (aka add "**a** subset" and "nodes"), "shown in subfigures **a**, **c**, **e**, **g**" (without "**b**"), "subfigure **b**, **d**, **f**, **h**" (with "**b**"). The left or upper figure of each couple contains the drought tolerance states of susceptibility, moderate tolerance and tolerance with intermediate levels (shown in subfigures **a**, **b**, **c**, **e**, **g**). The right or lower picture of each couple shows the populations and races identified for wild (subfigures **d**, **f**, **h**) and cultivated (subfigures *b, h*) common bean. For *Asr1*, the analysis was carried for the cultivated and the wild collections independently (subfigures a-b and c-f, respectively), and considered the exon and intron regions separately (subfigures *c-d* and *e-f*, respectively). For *Asr2*, the analysis is considered globally. Abbreviations are: Mesoamerican (M1 and M2), Durango (D1 and D2), Guatemala (G), Nueva Granada (NG1 and NG2) and Peru races (P1), and Mesoamerican (M_w), Guatemala (G_w), Colombian (C_w), Ecuador-Northern Peru (ENP_w) and Andean wild populations (A_w).

Globally, *Asr2* presented more haplotypes than *Asr1*. Moreover, haplotypes with low frequency were more common at *Asr2* than at *Asr1* (Table 4). A hypothetical haplotype was required to analyze variation in the exon region of *Asr1*. Furthermore, some patterns suggested by the previous section were revealed by total haplotype frequency for each gene pool division was clearer for *Asr1*, as well as for each sub-population (Figure 3). In particular, Northern Peru population had a unique fixed haplotype of *Asr1*. The haplotypes with the highest frequency were shared by accessions from Mesoamerican, Guatemala, Colombia and Andean populations for *Asr1* and *Asr2* genes. A pair of equally high-frequency haplotypes shared by more than two populations was found for *Asr2*. Interestingly, Ecuador-Northern Peru population did not have any of these haplotypes. A Mesoamerican-Andean gene pool division was clear for both genes, especially for *Asr1*, as was stated in the previous section.

**Table 4:** Haplotype information for SNPs across the 401 genotypes of common bean wild and Core collections

**Gene**	**Region**	**Haplotype name**	**Haplotype sequence**	Frequency*
*Asr1*	Cultivated	1	T	**56.40%**
		2	C	**43.60%**
	Wild intron	1	AC	**12.10%**
		2	CT	9.10%
		3	AT	**78.80%**
	Wild exon	1	TCC	2.70%
		2	CCT	**16.20%**
		3	CTC	**81.10%**
*Asr2*	Total	1	TGC	**85.20%**
		2	ATC	**12.70%**
		3	ATG	1.70%
		4	TGC	0.30%
	Cultivated**	1	ATG	**93.30%**
		2	TAT	6.70%
	Wild**	1	GCA	**93.30%**
		2	CAT	6.70%

*In bold: frequencies values bigger than 10%.**Haplotypes only observed in heterozygotes are not depicted.

**Figure 4:**
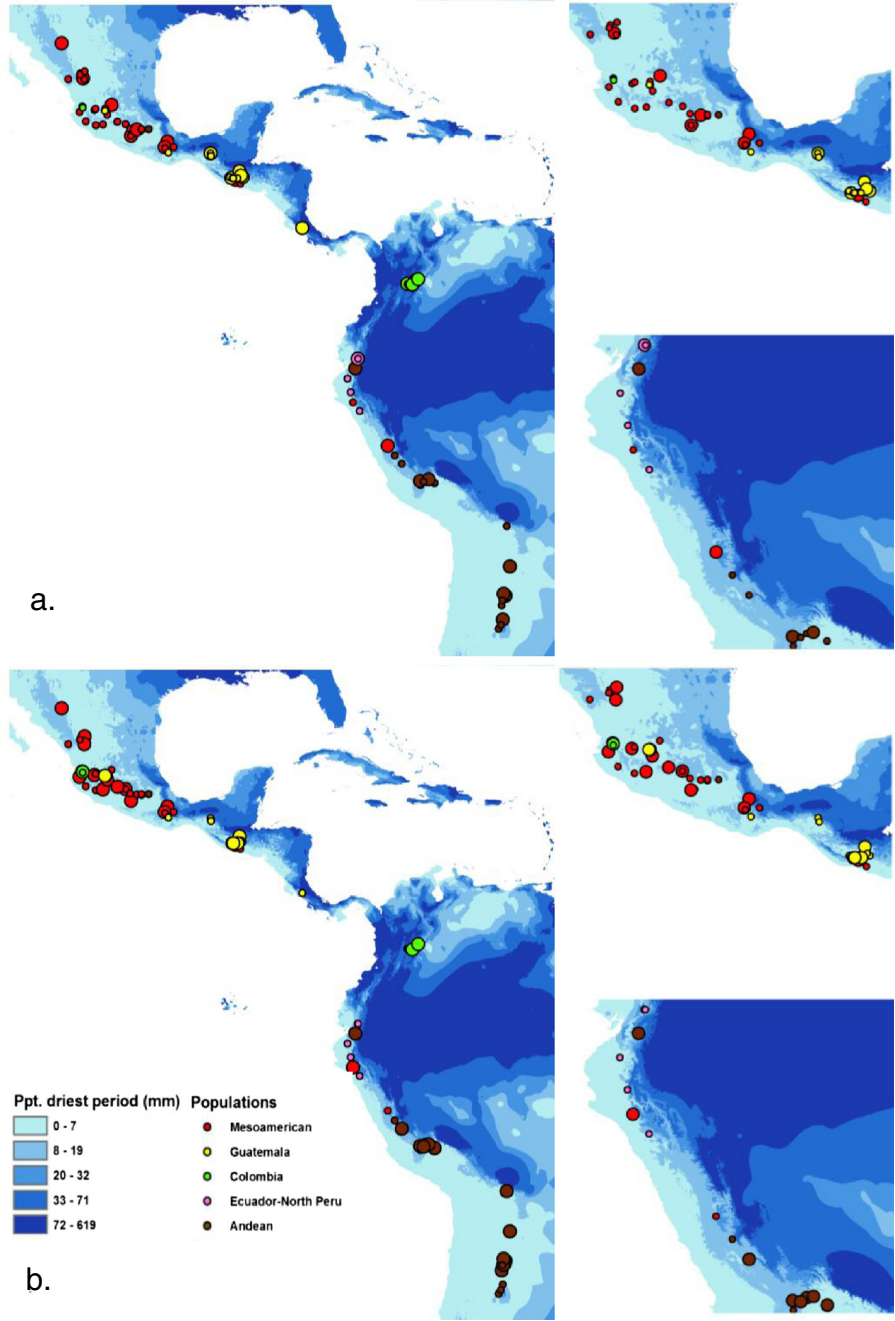
**Geographic distribution of wild common bean accessions.** Geographic distribution of wild common bean accessions considering genetic polymorphism and precipitation of driest period for **a**) *Asr1*_161 (in significant linkage disequilibrium with *Asr1*_233) and **b**) for the two major haplotypes of *Asr2*. Different circle sizes correspond to different allele or haplotypes. Mexico and Ecuador-Northern Peru regions are detailed in each case.

A qualitative evaluation of the possible association between population structure, drought tolerance, and haplotype and nucleotide diversity of candidate genes was made by comparing the allelic diversity with ecological variation for drought tolerance (Figure 4). Median Joining Networks aided in this inspection in this way, it was possible to identify genetic variation correlated with estimated habitat drought index and drought severity index for the wild and cultivated genotypes, respectively. In particular, the combination of estimated habitat drought stress, drought severity index, population structure, and candidate gene haplotypes in a qualitative analysis revealed four main categories of relationships, as described below.

First, some haplotypes included sequences of accessions from the same wild population but with very different estimated levels of drought stress associated with their habitat, as was the case for some *Asr2* haplotypes. Second, some haplotypes included accessions from dissimilar populations that presented a similar estimated habitat drought stress. Third, some accessions had distinct haplotypes but were categorized in a similar rank of estimated drought stress, irrespective of their populations. Fourth and finally, some accessions of the same population had dissimilar haplotypes and disparate drought stress ranks. Visual inspection of allelic variation for these haplotypes along different ecological regions confirmed the previous categories.

## Discussion

Common bean *Asr* genes are an example of a small gene family with a simple structure and potential role in drought tolerance. This paper is the first attempt to characterize their diversity in common bean. Furthermore, this research integrates different lines of evidence from the coalescent theory and co-evolution analysis to evaluate the role of these genes in drought tolerance. One of our major successes was to find that wild common bean is a reservoir of genetic variation at *Asr* genes, which may be useful for breeding drought tolerance in cultivated common bean.

### Wild common bean is a reservoir of genetic variation at *Asr* genes

Traditionally, wild relatives of cultivated plants were not subjected to bottlenecks or selective sweeps during the domestication process, and were not selected subsequently to improve specific traits. Hence, wild gene pools have not been genetically eroded and they conserve much of the original variation developed over millennia for the species [47]. Moreover, they are better adapted to some abiotic stress conditions from their original habitat than cultivars and they present higher levels of exogamy than cultivated accessions. Such adaptations were lost in the transition toward domestication and to field production, and gave way to a superior allocation of plant resources and biomass to yield. Consequently, wild relatives must have higher genetic diversity and phenotypic variability than cultivated individuals, except in the case of diversifying selection that were part of the domestication syndrome (set of traits that were selected during domestication [48]) in the crop.

This hypothesis of higher wild versus cultivated diversity has been demonstrated in rice [14,49], common bean [28,50], dogs [51,52], chickens [53], fishes [54], and many other species. The hypothesis of dissimilar grades of variation between wild and cultivated plants has been reinforced for common bean in the present research using the *Asr* candidate genes instead of genomic variation as previous studies have done. The candidate gene approach allows comparisons of adaptive variation to be made, and not just neutral polymorphism. This analysis of adaptive variation closes the gap between diversity analysis and functional genomics. Furthermore, the selective and population-based hypotheses used to explain the lack of neutrality are distinguished easily when adaptive and neutral variation is compared in a common framework. As we will discuss extensively in the following sections, a combination of SNP markers at *Asr* genes and SNP markers at a genome-wide scale, allowed us to conclude dissimilar diversity between wild and cultivated common beans. Different evolutionary imprints in each gene, and divergent correlation between candidate genes and drought tolerance, accounted in all cases for the evolutionary inertia and the confounding effects of population structure. In a first observation, we detected more genetic variation for drought tolerance in wild than in cultivated common beans. The variation found among the cultivated races was considerable, but lower than in wild common beans. Cultivated races span different regions across the Americas in relation with water stress. Therefore, drought tolerance is not expected to be uniform across the cultivated accessions, even though there was presumably not selection for drought tolerance during the domestication syndrome. In addition, some differences existed between the adaptation of wild and cultivated individuals to drought stress. The evaluation of drought physiology traits in wild populations of common bean would have been impractical due to long growth cycle and low biomass [55]. Hence, the ecological analysis we applied was predictive of drought tolerance.

### *Asr* paralogous have experienced different evolutionary patterns

Selective processes, such as purifying selection and local adaptation, imprint regions of the genome in different manners, causing the departure of genetic variation from neutral expectations [49,51]. Purifying selection is associated with low values of nucleotide diversity (π) and negative values of Tajima’s *D*. However, recent population bottlenecks tend to achieve the same reduction in nucleotide variation. On the other hand, local adaptation tends to homogenize haplotypes within the same niche, fix polymorphisms between different populations, and eliminate low frequency polymorphism. Consequently, few haplotypes with high frequency are generated, corresponding to high values of nucleotide diversity and global Tajima’s *D*. Nevertheless, independent domestication events, extensive population structure and population expansions after bottlenecks can also leave the same patterns [43]. In the case of cultivated common bean, at least two independent domestication processes, in the Andes and in Central America, generated extensive population structure and a genome-wide increase in the global nucleotide diversity [27,33]. That is why we have observed a significant positive, bimodal distribution of π values. However, this pattern was lost when the two gene pools were considered independently. It remains to be determined whether a population expansion after the two independent domestication bottlenecks could be relevant to explaining the observed patterns of gene diversity. We did observe significant population expansion for the Mesoamerican wild population using the Ramos-Onsins & Rozas’ R^2^ statistic [56]. We could not, however, find the same pattern for the Andean gene pool. We predict that an extensive survey of SNP markers will reinforce further conclusions. Particularly, a well saturated genome-wide mismatch distribution will allow us to confirm the extent of population expansion within Mesoamerican or Andean beans.

For wild common bean, the global neutrality test against the Wright-Fisher neutral model was rejected for *Asr2* showing a bias toward high nucleotide diversity. This was not the case for *Asr1*, where the neutrality test was rejected when made against the evolutionary background, showing a bias toward low nucleotide diversity. Moreover, when the gene pools were analyzed independently a significantly negative Tajima’s *D* was observed from the Wright-Fisher equilibrium for *Asr1* but not for *Asr2*. This behavior was likely to be a consequence of the fact that the Wright-Fisher neutral model did not account for the population structure or the evolutionary processes of the species, while the background distribution successfully captured the demographic complexity of common bean. Therefore, the evolutionary background of a crop like common bean is the ideal framework to make straightforward comparisons between candidate genes and genome-wide variation, and this is perfectly equivalent to applying the neutrality test to each one of the sub-populations, as well. Both sources of evidence undoubtedly suggest that *Asr1* was subjected to purifying selection at least in wild common bean.

This has been a common finding in other genes and species. For example, ABA related transcription factors in wild tomatoes [57], genes and chromosomes in maize [58,59], and genomic regions in chickens [53] and rice [60], have been associated with local or genomic selective sweeps. Indeed, the implications of these findings have been important because once one knows how selective processes have proceeded then one can identify this pattern elsewhere [61].

It is necessary to emphasize that population structure and climatic variability are partially correlated because both follow a latitudinal pattern, as was suggested previously [29,62]. This is particularly true for *Asr2* because its variation overlaps with the evolutionary background. Although population structure explains its significant high nucleotide diversity, there is not power to explain an association between *Asr2* and the estimated drought tolerance, if any.

The haplotype/allelic analysis accounted for population structure. Therefore, false positive associations that are especially common in haplotype – phenotype correlations can be rejected [63]. Local adaptation explains these discoveries because it imprints the genetic regions with global high nucleotide diversity, and is congruent with ecological (estimated drought tolerance) and genetic (population structure) characteristics as well. Neither population structure nor population expansions after bottlenecks explain *per se* and *en plene* both results. Rather it is more likely that purifying selection caused by ecological adaptation on wild sources of domesticates explains the observed variation between wild and cultivated genotypes. Similar patterns have been found in cattle [64]. Genomic signatures of local adaptation have been revealed in *Phytophthora infestans*[65] and *Brugia malayi*[66], and were proposed in trees, as well [67]. More specifically to our study, *Asr2* has been associated with adaptive variation for arid habitats in wild tomatoes [11,18]. Particularly, Fischer *et al.*[68] found that two genes of the same ASR family (associated with the ABA related drought response pathway) presented dissimilar patterns of nucleotide variation in wild tomatoes, accounting for purifying selection and local adaptation. Hence, our study is the second report of paralogous genes with the ABA domain that present quite different selection signatures in wild relatives of a crop.

Finally, we found signatures of purifying selection and local adaptation in the wild collection. This is a consequence of the enormous genetic and phenotypic variation stored in the wild populations. In addition, there were differential selection patterns between wild and cultivated beans, which explained why we did not detect selective signatures at the cultivated gene pool level, but we did at the wild gene pool level. Wild common bean is a viney annual plant that germinates among small trees and shrubs in forest clearings or in disturbed environments with the onset of seasonal rains [55]. Specifically, the growth cycle of the wild common bean in the tropics and subtropics is from 6 to 10 months in length, where with bimodal rainfall a mid-season dry period occurs that can last 2 to 4 weeks to as long as 3 months on the equator. In response to this mid-cycle drought the wild *P. vulgaris* enters a survival mode of slow growth and reduced physiological activity, until rainfall resumes and flowering occurs. In contrast, cultivated beans are not subjected frequently to these environmental pressures which may explain less selection for novel variability in drought related genes such as *Asr1* and *Asr2*.

In short, the adaptive importance of *Asr1* was inferred because we detected a distortion in its genetic pattern in relation with the evolutionary background. In the future, expression analysis and QTL information will be needed to validate the importance of *Asr1* in the context of drought tolerance in common bean. Overall, however, we have found a valuable gene for plant breeding in common bean and have also proven that signatures of selection are good predictors of markers and genes associated with a desired trait. The value of wild beans from dry environments to improve Andean or Mesoamerican cultivars should be explored empirically especially given that wild common beans occupy more geographical regions with extensive drought stress in temperate or high-subtropical zones than cultivated accessions. These regions include the arid parts of the Andes in Peru, Bolivia and Chile, or the infertile valleys of the southwestern Mexico. With this work we also lay the groundwork for the evaluation of wild and cultivated common bean collections to analyze other candidate genes for drought tolerance.

## Conclusions

This research suggested the importance of *Asr1* in the context of drought tolerance, and constitutes the first steps towards an association study between genetic polymorphism of this gene family and variation in drought tolerance traits. Furthermore, one of our major successes was to find that wild common bean is a reservoir of genetic variation and selection signatures at *Asr* genes, which may be useful for breeding drought tolerance in cultivated common bean.

## Methods

### Plant material

Totals of 104 wild common bean accessions and 297 cultivated accessions were considered in this study. These 401 accessions from reference and core collections were selected to be representative samples of the gene pools and races in the cultivated core collection analyzed by Blair *et al.*[5], and to be subsets of the wild bean core collection developed by Tohme *et al.*[24].

Control genotypes included the Andean genotypes Calima (G4494) and Chaucha Chuga (G19833), as well as the Mesoamerican genotypes ICA Pijao (G5773) and Dorado (DOR364), with common name and germplasm entry or advanced line name listed in each case in parenthesis. Seed samples for wild accessions were provided by the Genetic Resource Unit ( http://isa.ciat.cgiar.org/urg/main.do), while the cultivated reference collection is maintained by the bean program from original stock at the same Unit.

### DNA extraction, primer design, DNA amplification and sequencing

Total DNA was extracted in the Germplasm Characterization Laboratory of CIAT [69]. The primers used to amplify the three regions of the *Asr1* and *Asr2* genes were provided by the ADOC project or designed from ASR gene sequences (Table 5). Amplification conditions for genes *Asr2* and *Asr1* (region 1 and 2) used thermocycling conditions of a 95°C hot start for 5 min, followed by 35 cycles of 95°C denaturation for 1 min, annealing for 1 min at 62°C, 53°C or 60°C for the *Asr1* region 1, *Asr1* region2, and *Asr2* gene, respectively. All PCR reactions were followed by a 72°C extension for 2 min.

**Table 5:** **Primers used for PCR amplifications of candidate genes in *P. vulgaris *in the Core and wild collections **

**Gene**	**Primer Name**	**F/R**	**Sequence (5′-3′)**	**Ta (°C)**	**Source**	Sequence*
*Asr1* (region 1)	ADOC01_01_Pv_12	F	GAGGAGACTAAGCCCATAGA	62	IRD	TC2798
	ASR_TC2798_RV**	R	TGGACAGAAGCCATTCACTCCCAA		CIAT	
*Asr2* (region 2)	ASR_TC2798_FW2	F	AAGCACCACAAGCATCTTGAGCAC	53	CIAT	
	**	R	**		**	
*Asr2*	ADOC01_03_Pv_05	F	CCACCACCACAAAGAGGA	60	IRD	CA910244
	ADOC01_03_Pv_06	R	CAAACATTCTTCAAACTTGCTCAGA		IRD	

*Accession ID of the original EST sequences used to design the primers. TC accessions are from Gene Index (compbio.dfci.harvard.edu/tgi/).**The reverse primer ASR_TC2798_RV was also used in this case.

The PCR reactions were carried out in a 25 μl final volume containing 65 ng of genomic DNA, 1 X PCR buffer (1 X: 10 mM of Tris–HCl pH 8.8, 50 mM of KCl, 0.1% of TritonX-100), 0.3 μM (for the *Asr1* - region 1) or 0.5 μM (for the *Asr1* - region 2 and *Asr2* gene) of each of the forward and reverse primers; 2 mM of MgCl_2_; 0.4 mM of total dNTPs; and 1 U (for the *Asr1* – region 1), 1.25 U (for *Asr2*), or 2 U (for the *Asr1* – region 2) of *Taq* Polymerase (Fermentas).

Size determination of PCR products was carried out by gel electrophoresis through a 1.5% agarose gel using Tris-Borate-EDTA buffer containing SYBR-Green. PCR products were purified using Exo-*Sap* clean-up reactions. These clean products were used as templates for subsequent Sanger sequencing reactions, using BigDye Terminator v3.1 Cycle Sequencing Kits. The samples were run on an ABI prism 3730 automated sequencer at the Cornell University Biotechnology Resource Center. Base-pair calls, quality score assignment, and construction of contigs were carried out using Sequencher 4.7 (Gene Codes Corp., Ann Arbor, Michigan). Sequences have been deposited at GenBank under accession numbers JX082400 - JX083058.

### Gene characterization, domain detection, and protein and nucleotide alignments

Exons and introns were determined through Blastn of the sequenced regions against the well characterized soybean genes and the non-redundant (nr) EST database with a gap open penalty of 5, a gap extension penalty of 2, a match score of 2, and a mismatch score of −3. Coding regions, UTRs, reading frames and conserved domains were determined through Blastx of the new sequences against nr protein database with a gap open penalty of 11, a gap extension penalty of 1 and a BLOSUM62 matrix available from http://blast.ncbi.nlm.nih.gov.

The ASR domain region was confirmed using the Pfam website ( http://pfam.sanger.ac.uk/). Nucleotide and protein alignments, as well as Neighbor-Joining trees, were constructed using orthologous and paralogous genes to verify conserved regions. Nucleotide alignments were carried out with MUSCLE algorithm [70] and Geneious 4.0 software (Biomatters Ltd.). The sequences were also manually examined to verify the quality of the alignment. Haplotype reconstruction was carried out using PHASE software [71].

### Patterns of nucleotide diversity

Allele assignments and frequencies for all the accessions were used to calculate the polymorphic information content (PIC) for each SNP marker according to Anderson *et al.*[72] using the formula ${\text{PIC}}_{i}=1\;-\;\Sigma p_{\text{ij}}^{2}$; were p_ij_ is the frequency of the allele *j* for each marker *i*. Levels of genetic diversity within domesticated and wild common bean were quantified with measures of nucleotide diversity based on the number of segregating sites (θ_W_) [73] and based on the average number of nucleotide differences per site between sequences (π) [74]. These calculations were carried out with the program DnaSP 5.10 [75]. *G*_*ST*_, *F*_*ST*_ and Snn values were also computed with the same software. Meanwhile, the number of haplotypes and the haplotype diversity (H_d_) were calculated with the same software. Median joining haplotype networks were built using Network 4.5.1 [76].

Neighbor Joining tree construction and nodal support evaluation using 1000 bootstrap replicates were carried out with the program Mega4 [77]. Network trees accounted for population subdivision based on K-values for number of subpopulations as determined by Blair *et al.*[5]. Drought tolerance for each genotype was based on geographic origin and estimated habitat drought index (according to the formula [PET-P]/PET, where PET and P accounts for potential evotranspiration and precipitation, respectively. PET was calculated following two different approaches based on temperature and radiation [78,79]) or field testing and an actual drought severity index (according to the index of Rosales *et al.*[80] applied on 8 × 8 and 12 x 12 lattice trials with three repetitions each and two environments - drought and irrigation, evaluated at 2008 following the same methodology reported by Blair *et al.*[4]) for wild and cultivated accessions, respectively [81].

Tests for selection were performed to estimate whether *Asr1* and *Asr2* followed the Wright-Fisher model of neutral evolution in each subpopulation. Tajima’s *D*[82] tests were carried out with DnaSP using 5,000 coalescent simulations [43] also for each division of wild vs. cultivated beans, Andean vs. Mesoamerican gene pools and race found in each group. Moreover, the nucleotide diversity of Nei was contrasted against an estimated distribution (evolutionary background) using random gene based-SNP markers unrelated to drought stress [46]. Such comparison took into account the differences between markers in terms of number of alleles and information content, as well as the basics of population structure in common bean [46]. The results obtained with the neutral model were compared with the ones obtained using the background distribution in order to access the power of the latter to capture the complexity of demographic processes in common bean.

## **Authors’ contributions**

AJC and CMC carried out laboratory work, genetic and statistical analyses. SM contributed with the statistical analysis. MWB and DT participated in the design and coordination of the study. AJC and MWB drafted the manuscript. All authors read and approved the final manuscript.
